# The prevalence of alpha-1 antitrypsin deficiency in Ireland

**DOI:** 10.1186/1465-9921-12-91

**Published:** 2011-07-13

**Authors:** Tomás P Carroll, Catherine A O'Connor, Olwen Floyd, Joseph McPartlin, Dermot P Kelleher, Geraldine O'Brien, Borislav D Dimitrov, Valerie B Morris, Clifford C Taggart, Noel G McElvaney

**Affiliations:** 1 Department of Medicine, Royal College of Surgeons in Ireland Education and Research Centre, Beaumont Hospital, Dublin 9, Ireland; 2 Trinity Biobank, Institute of Molecular Medicine, St James's Hospital, Dublin 8, Ireland; 3 Department of General Practice, Royal College of Surgeons in Ireland, 123 St. Stephens Green, Dublin 2, Ireland; 4 School of Medicine and Dentistry, Queens University Belfast, Northern Ireland

## Abstract

**Background:**

Alpha-1 antitrypsin deficiency (AATD) results from mutations in the SERPINA1 gene and classically presents with early-onset emphysema and liver disease. The most common mutation presenting with clinical evidence is the Z mutation, while the S mutation is associated with a milder plasma deficiency. AATD is an under-diagnosed condition and the World Health Organisation recommends targeted detection programmes for AATD in patients with chronic obstructive pulmonary disease (COPD), non-responsive asthma, cryptogenic liver disease and first degree relatives of known AATD patients.

**Methods:**

We present data from the first 3,000 individuals screened following ATS/ERS guidelines as part of the Irish National Targeted Detection Programme (INTDP). We also investigated a DNA collection of 1,100 individuals randomly sampled from the general population. Serum and DNA was collected from both groups and mutations in the SERPINA1 gene detected by phenotyping or genotyping.

**Results:**

The Irish National Targeted Detection Programme identified 42 ZZ, 44 SZ, 14 SS, 430 MZ, 263 MS, 20 IX and 2 rare mutations. Analysis of 1,100 randomly selected individuals identified 113 MS, 46 MZ, 2 SS and 2 SZ genotypes.

**Conclusion:**

Our findings demonstrate that AATD in Ireland is more prevalent than previously estimated with Z and S allele frequencies among the highest in the world. Furthermore, our targeted detection programme enriched the population of those carrying the Z but not the S allele, suggesting the Z allele is more important in the pathogenesis of those conditions targeted by the detection programme.

## Introduction

Alpha-1 antitrypsin (AAT) deficiency is a hereditary disorder first reported in the early 1960s when emphysema was described in patients with low plasma levels of AAT protein [1]. The condition is associated with substantially increased risk for the development of pulmonary emphysema by the third or fourth decades of life and is also associated with risks for development of hepatic disease [2], cutaneous panniculitis [3], bronchiectasis [4], vasculitis [5], Wegener's granulomatosis [6], and lung cancer [7]. AAT deficiency is characterised by misfolding of the AAT protein and belongs to a class of genetic diseases termed conformational disorders [8].

The SERPINA1 gene is highly pleiomorphic with over 100 alleles identified to date [9]. Mutations which confer an increased risk of developing pulmonary emphysema and/or liver disease are those in which deficiency alleles are combined in homozygous or heterozygous states, yielding AAT serum levels below a putative protective threshold of 11 μM. The most common variants associated with disease are the Z (Glu342Lys) and S (Glu264Val) mutations, caused by a single amino acid replacement of glutamic acid at positions 342 and 264 of the polypeptide, respectively [8]. The class of SERPINA1 variants termed "null" mutations lead to a complete absence of AAT production and while extremely rare, confer a particularly high risk of emphysema [10].

AATD is an under-diagnosed condition with most cases misdiagnosed as COPD or non-responsive asthma. As a result, long delays between presentation of first symptoms and correct diagnosis are commonplace [11]. Guidelines issued by both the World Health Organisation and the American Thoracic Society/European Respiratory Society (ATS/ERS) recommend the establishment of targeted screening programmes for the detection of patients with AATD [12,13]. Moreover, while a large number of cohorts have been investigated, many of these studies were based on screening symptomatic patients, and performed on small groups of less than 500 individuals with accompanying high risk of error.

Apart from a few notable exceptions, such as a Swedish neonatal screening study [2], the lack of large population based studies using random sampling means the true prevalence of AATD in most European countries remains unknown. To address the paucity of data relating to AATD in the Irish setting, we analysed 1,100 individuals taken at random from the general population. In addition, we analysed a targeted population of symptomatic individuals and compared the findings with our general population to investigate whether targeted detection increased yield across all deficiency allele groups.

## Materials & Methods

### Subjects

**Figure 1:**
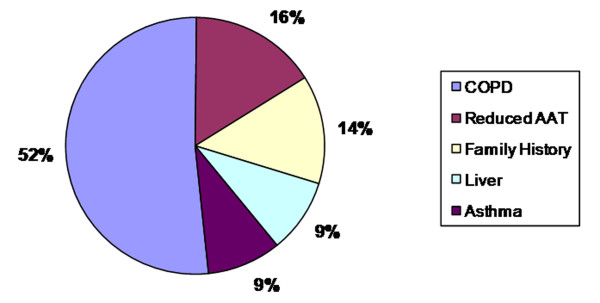
**Percentage distribution of 3,000 INTDP patients according to reason for screening**.

A total of 3,000 individuals were screened as part of the Irish National Targeted Detection Programme (INTDP). The detection programme is ongoing and began in May 2004 supported by funding from the Irish Government. The criteria for targeted screening were COPD, non-responsive asthma, cryptogenic liver disease, first degree relatives of known AATD patients (including ZZ, SZ and MZ) and individuals with reduced serum AAT levels according to the joint ATS/ERS guidelines (Figure 1). In addition, 1,100 individuals were screened from the Trinity Biobank DNA collection at St. James's Hospital Institute of Molecular Medicine, Dublin. The Trinity Biobank is a national buccal swab DNA collection selected at random from the electoral register.

### Quantification of AAT

AAT levels were measured by radial immunodiffusion (RID) (Siemens) or by nephelometry (Dade-Behring BN II). It must be noted that discrepancies exist when comparing these two methods for serum AAT quantification. Nephelometric methods can overestimate AAT concentrations due to haemoglobin or lipid interference, while RID-based methods have been shown to overestimate AAT concentrations by as much as 35-40% [14] and are less precise than nephelometric methods with higher coefficients of variation [15]. Moreover, the lower sensitivity inherent to the RID method because of the high lower limit of detection (0.33 g/L) becomes a factor when testing ZZ individuals with AAT concentrations <0.33 g/L.

### Phenotyping

**Figure 2:**
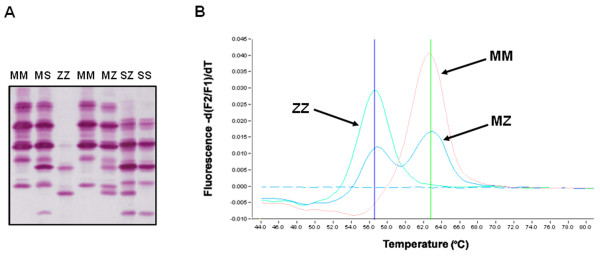
**Methods employed for analysis of AAT mutations**. (A) Typical isoelectric focusing gel used for identification of AAT phenotype. (B) Genotyping assay used to identify the Z mutation.

Qualitative detection and characterisation of AAT phenotypes was carried out using the Hydrasys electrophoresis platform (Sebia) and the Hydragel 18 A1AT Isofocusing kit (Sebia)(Figure 2A) [16]. This isoelectric focusing (IEF) method on agarose gel has an added immunofixation step which utilises a specific antibody to AAT, rendering it superior to traditional IEF techniques.

### Genotyping

Genotyping was performed on a LightCycler 480 (Roche) with specific primers and probes (Metabion) designed for the Z and S mutations as described in a previous publication (Figure 2B) [17].

### Data elaboration and statistical analysis

The prevalence and numbers of genotypes in the Irish population was calculated by applying the Hardy-Weinberg principle. Frequencies of genotypes and phenotypes were calculated and a derivative parameter, notably the type and number of mutations in each database, defined (Z, S, etc.). Data was analysed by descriptive statistics, percentage distribution, chi-square tests and methods for calculating odds ratios (ORs), 95% confidence intervals (95% CIs), accuracy and other contingency parameters, as appropriate [18]. Statistical significance was assumed at two-tailed p < 0.05, unless stated otherwise.

## Results

### Frequency of Z and S alleles in a random sample of the Irish Population

**Figure 3:**
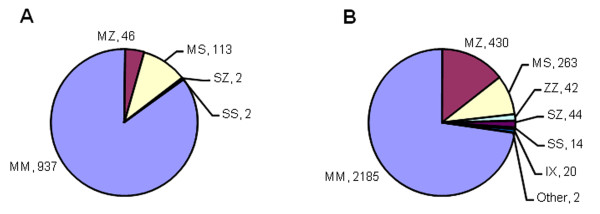
**Analysis of AAT mutations in Ireland**. (A) 1,100 DNA samples in the Biobank collection were genotyped for the S and Z mutations. (B) 3,000 Irish individuals were screened as part of the national targeted detection programme following ATS/ERS guidelines.

In the Trinity Biobank collection, 113 MS heterozygotes, 46 MZ heterozygotes, 2 SS homozygotes, and 2 SZ compound heterozygotes were identified (Figure 3A). This data yields a frequency of 0.0218 for the Z allele and 0.0541 for the S allele in the Irish population. Assuming Hardy-Weinberg equilibrium and based on a population of 4.24 million inhabitants (Census of Ireland 2006, http://www.cso.ie) these allele frequencies yield 2,015 ZZ individuals, 10,001 SZ individuals and 12,409 SS individuals (Table 1). Thus, the estimated prevalence of severe AATD (ZZ homozygotes) in Ireland is 1/2,104. In addition to ZZ AATD, the estimated prevalence of intermediate AATD (SZ compound heterozygote) is 1/424, with this phenotype also at increased risk of lung and possibly liver disease, while the estimated prevalence of mild AATD (SS homozygote) is 1/341. Finally, in terms of carriers, the calculated Z and S allele frequencies yield 170,832 MZ heterozygotes and 423,947 MS heterozygotes with an estimated prevalence of 1/25 for MZ and 1/10 for MS.

**Table 1:** Estimated Prevalence of AAT Genotypes in Ireland

Genotype	Prevalence [%, 95% CI]	Numbers in Ireland
**MS**	1/10 [10.00%, 9.70 - 10.30%]	423,947
**MZ**	1/25 [4.03%, 3.97 - 4.09%]	170,832
**SS**	1/341 [0.29%, 0.20 - 0.40%]	12,409
**SZ**	1/424 [0.24%, 0.23 - 0.25%]	10,001
**ZZ**	1/2,104 [0.05%, 0.04 - 0.06%]	2,015

Data from the Trinity Biobank presented as prevalence [% of total population, 95% confidence interval (CI)]. These figures are based on an Irish population of 4.24 million in the Republic of Ireland (www.cso.ie).

### Prevalence of AATD in the Irish National Targeted Detection Programme

A total of 3,000 individuals screened for AATD as part of the national targeted detection programme identified 430 MZ heterozygotes, 263 MS heterozygotes, 44 SZ compound heteroygotes, 42 ZZ homozygotes, and 14 SS homozygotes (Figure 3B), with allele frequencies of 0.0938 for the Z mutation and 0.0518 for the S mutation in the targeted population. A further 20 individuals with the I mutation (Arg39Cys), associated with a mild plasma deficiency [19] similar to the S mutation, were identified with an allele frequency of 0.0033, as well as two individuals with extremely rare mutations, V [20], and Z_bristol _[21]. Serum AAT levels among the various phenotypic groups illustrate the relationship between decreasing AAT levels and increasing risk of disease (Table 2).

**Table 2:** AAT Deficient Phenotypes and Concentrations in the INTDP

Phenotype	N	Mean AAT (g/L +/- SEM)	AAT range (g/L)
**MV**	1	1.28	n/a
**MI**	12	1.285 +/- 0.095	0.93 - 2.08
**MS**	263	1.199 +/- 0.021	0.499 - 3.32
**MZ**	430	0.871 +/- 0.014	0.442 - 4.08
**IS**	3	1.004 +/- 0.103	0.801 - 1.14
**SS**	14	0.842 +/- 0.046	0.556 - 1.20
**IZ**	5	0.605 +/- 0.076	0.333 - 0.801
**SZ**	44	0.564 +/- 0.021	0.23 - 0.98
**Z/Zbristol**	1	0.50	n/a
**ZZ**	42	0.11 +/- 0.012	0.05 - 0.333

AAT deficient phenotypes and AAT concentrations from the first 3,000 individuals screened in the INTDP listed according to increasing risk of disease. Data presented as mean AAT concentration +/- standard error of the mean (SEM).

### Comparison of Z and S allele frequencies

**Figure 4:**
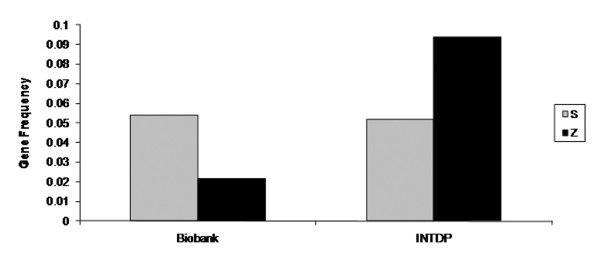
**Comparison of S and Z allele frequencies in the Biobank population and in the INTDP population**.

The frequency of the S allele in a random sample from the general population was similar to that identified in the targeted population. However, the frequency of the Z allele was four-fold higher in the targeted population compared to the general population (Figure 4). The numbers of Z and S alleles in both populations are presented in Table 3. The risk of being registered with a specific condition in the INTDP database is 4.6 times higher in subjects carrying the Z mutation than in other carriers or non-carriers (OR = 4.64, 95% CI 3.41 - 6.19). This is most likely due to the increased risk associated with the Z mutation compared to the S mutation alone (OR = 4.48, 95% CI 2.88 - 5.92).

**Table 3:** Comparison of the Distribution of S and Z Alleles in the INTDP cohort and the Biobank cohort

Phenotype	INTDP	Biobank	Total
**S versus other**			
S alleles	311	119	430
Other (including Z)	5689	2081	7770
Total alleles	6000	2200	8200
**Z versus other (p < 0.001)**			
Z alleles	563	48	611
Other (including S)	5437	2152	7589
Total alleles	6000	2200	8200
**Z versus S (p < 0.001)**			
Z alleles	563	48	611
S alleles	311	119	430
Total alleles	874	167	1041

Data analysed by chi-square test with Yates correction.

## Discussion

Our study describes the prevalence of AATD in a randomly selected sample of 1,100 individuals from the Irish population and in a targeted population, specifically with regard to the two most common alleles associated with AATD, the Z and S mutations. In the general Irish population the Z mutation occurs at a frequency of 0.0218, while the S mutation occurs at a frequency of 0.0541. This means 1 in 25 Irish individuals are heterozygous for the Z allele and 1 in 10 are heterozygous for the S allele. More importantly from a clinical perspective, 1 in 2,104 Irish individuals are ZZ homozygotes, 1 in 424 are SZ compound heterozygotes, and 1 in 341 are SS homozygotes. In comparison, when we looked at the INTDP population the Z mutation occurred at a frequency of 0.0938 and the S mutation occurred at a frequency of 0.0518. This means that 1 in 7 tested were MZ and 1 in 11 tested were MS. Strikingly, 1 in 71 tested were ZZ, 1 in 68 were SZ and 1 in 214 were SS. Taken as a whole, 27.1% of the targeted population contained at least one AAT mutation.

Comparing data from the two groups investigated, the allele frequency for Z is over four-fold higher in the targeted population compared to a sample of the general population. Targeted detection programmes produce a higher rate of AATD detection although the risk of missing asymptomatic individuals exists. However, the four-fold increase in Z frequency observed highlights the effectiveness of the targeted screening approach as advocated by ATS/ERS as the most cost-effective method of detection [12]. There is particular emphasis in the INTDP on aggressive family screening to identify asymptomatic relatives before significant lung damage has occurred. This increases the rate of detection of deficient alleles, and in our study 14% of INTDP individuals were screened because of a deficient first-degree relative. Nonetheless, the high frequency of the Z allele in the targeted group underlines the role of this mutation in the pathogenesis of lung and liver disease. In contrast to Z, the allele frequency for S was not significantly increased in the targeted population compared to the Biobank cohort. This finding was not surprising considering the lack of strong evidence for risk of disease due to the S mutation. The S allele is associated with a mild plasma deficiency of AAT as the S AAT protein is less polymerogenic than Z AAT [19], and the S allele is assumed clinically significant only when co-inherited with Z or other deficient alleles. For example, the SZ genotype is a significant risk factor for COPD [22,23]. Evidence also exists of abnormal liver function as characterised by elevated liver enzymes in newborn SZ individuals [2] and a possible risk for liver disease in later life [24,25]. However, a risk of COPD due to the MS genotype was not found [23].

A previous Irish study investigated the prevalence of AATD in 111 Irish coeliac patients compared to 250 blood donors and found gene frequencies of 0.008 for Z and 0.04 for S in the blood donor group [26]. However, the small sample size and the less accurate isoelectrofocusing method employed may account for the discrepancies with our findings. We have previously used the same phenotyping method and found the MZ phenotype was often difficult to correctly identify, compared to the more accurate and reliable Sebia method now used in our laboratory. Similar to the revised Irish data on AATD presented here, studies in other countries may have significantly under-estimated the frequency of the Z and S mutations due to small sample size and/or methodological limitations.

Throughout Europe the frequency of the Z and S mutations varies widely between countries, geographic regions, and ethnic groups. Approximately 3 - 4% of northern Europeans carry the Z allele and 6% carry the S allele [27]. The highest frequency of the S allele is found in the Iberian Peninsula with a mean gene frequency of 0.0564, suggesting the mutation is likely to have arisen in the region. Placing our results in a European context, we observe that the frequency of 0.0541 for the S mutation in Ireland is among the highest in Europe, and similar to the Iberian Peninsula. The frequency of the Z variant is highest in northern and western European countries with a mean gene frequency of 0.014, peaking in southern Scandinavia with a gene frequency of >0.02 [28]. Similar to the S allele, the frequency of 0.0218 for the Z allele in the Irish population is also among the highest in Europe. Interestingly, as the genotyping methods employed to study the Biobank cohort only identify Z and S alleles it is worth considering the gene frequencies described may still underestimate AATD in Ireland. Other more rare SERPINA1 mutations could also be present in the Biobank cohort, for example, we identified 22 rare non-Z, non-S mutations in the INTDP cohort which would be missed by our genotyping method.

The high prevalence of AATD in Ireland is not without precedent. Ireland has the highest prevalence of cystic fibrosis [29,30] and haemochromatosis [31] in Europe, as well as high frequencies of other genetic diseases [32]. This can be partly explained by the geographical isolation of an island on the fringes of Western Europe, with the genetic background of the population remaining largely undisturbed by the demographic movements that prevailed on mainland Europe. The Z mutation is thought to have arisen from a single origin 66 generations or 2,000 years ago [33,34], and its high frequency in southern Scandinavia suggests that the mutation arose in this area and was subsequently dispersed by migration patterns such as the Viking colonisation of north-western Europe between 800 and 1200 AD [35]. The relatively high frequency of the Z allele in the Irish population may represent a Viking genetic footprint resulting from significant settlement in Ireland in the period from 800 to 1200 AD when large towns and urban centres were established by Viking settlers including modern Dublin, Limerick and Cork [36]. The long history of emigration from Ireland would also suggest that populations of Irish descent in countries such as America, Canada, and Australia contain high frequencies of the Z mutation (and S mutation) and may benefit from screening for AATD.

The relatively high frequency of the S mutation could suggest that the tribes who first settled on Irish shores may have migrated from the Iberian Peninsula. The S mutation is older than the Z and is postulated to have arisen in the north of the Iberian Peninsula and subsequently spread throughout Europe during mass migration [37]. For example, one of the highest reported frequencies of the S allele in Europe is in the region of Galicia in north-western Spain [38], and in general high S frequencies are found all along the western Atlantic seaboard [28]. Other genetic similarities have been described that suggest a shared ancestral heritage among the populations on the Atlantic façade of Europe, stretching from northern Iberia to western Scandinavia and dating back to the end of the last Ice Age [39].

Another intriguing theory postulated to explain the high prevalence of AATD in European populations is the Z and S mutations confer a survival advantage on heterozygotes, of particular relevance in the pre-antibiotic era [40]. Polymers of Z AAT protein have been found in lung lavage and shown to act as neutrophil chemoattractants [41], and an enhanced inflammatory response has been demonstrated in MZ heterozygotes [42]. The proposed hypothesis suggests the Z and S alleles favour the generation of polymers at sites of inflammation and these polymers help focus and amplify the host inflammatory response to eradicate invading infectious organisms.

In summary, the findings of our study have significant consequences. We show that AATD is more prevalent than previously estimated in Ireland [28], with over 2,000 ZZ and 10,000 SZ individuals at significantly increased risk of developing lung and liver disease. A further 170,000 MZ heterozygotes are estimated in the Irish population and this group may also be at risk of developing COPD, particularly in individuals who smoke [43]. Moreover, we reaffirm the importance of the Z allele in the clinical disorders associated with AATD. The INTDP enriched the population of those carrying the Z but not the S allele, suggesting the Z allele is more important in the pathogenesis of those conditions targeted by the detection programme.

It is clear from the data presented here that the statement "AATD is not a rare disease but a disease that is rarely diagnosed" is particularly apt in the Irish setting [44]. The continuing lack of awareness and under-diagnosis of this condition is alarming considering the high numbers of individuals at risk due to deficient SERPINA1 mutations. The advantages of early and accurate diagnosis of AATD are manifold and include closer observation and management of affected individuals, especially regarding pulmonary and liver health; family member testing; aggressive smoking cessation efforts; consideration of occupational hazards and environment exposures; and significant economic benefits arising from the reduced burden on healthcare providers [11,45].

## Conclusion

This study demonstrates that the Z and S allele frequencies in Ireland are among the highest in the world, with large numbers of individuals at risk of disease due to AATD in the Irish population. The vast majority of these individuals remain undetected. The importance of an early diagnosis of AATD cannot be over-emphasised as the resulting appropriate medical follow-up and lifestyle changes can help prevent or at least postpone the development of the lung and liver disease associated with this condition.

## List of abbreviations

AAT: alpha-1 antitrypsin; AATD: alpha-1 antitrypsin deficiency; ATS/ERS: American Thoracic Society/European Respiratory Society; COPD: chronic obstructive pulmonary disease; IEF: isoelectric focusing; INTDP: Irish National Targeted Detection Programme.

## Competing interests

TPC received the eALTA award 2007 from Talecris Biotherapeutics. The other authors declare they have no competing interests to disclose.

## Authors' contributions

TPC contributed to the conception and design of this study, acquisition of data, analysis and interpretation of data and manuscript preparation. COC contributed to the acquisition of data, drafting and revision of the manuscript and final preparation. OF contributed to the acquisition of data and analysis and interpretation. JMcP and DPK contributed to the acquisition of data, drafting and revision of the manuscript and final preparation. GOB contributed to the acquisition of data, analysis and interpretation of data and manuscript preparation. BDD contributed to the statistical analyses of the data and manuscript preparation. VBM contributed to the acquisition of data and manuscript preparation. CCT contributed to the acquisition of data, methodologies employed, and manuscript preparation. NGMcE contributed to the conception and design of this study, drafting and revision of the manuscript and final preparation. All authors read and approved the final manuscript.
